# Colorimetric Immunoassays with Boronic Acid-Decorated, Peroxidase-like Metal-Organic Frameworks as the Carriers of Antibodies and Enzymes

**DOI:** 10.3390/molecules29133000

**Published:** 2024-06-24

**Authors:** Ting Sun, Xinyao Yi, Lin Liu, Feng Zhao

**Affiliations:** 1 Guizhou Provincial University Key Laboratory of Advanced Functional Electronic Materials, School of Chemistry and Materials Science, Guizhou Education University, Guiyang 550018, China; 2 College of Chemistry and Chemical Engineering, Central South University, Changsha 410083, China; 3 College of Chemistry and Chemical Engineering, Anyang Normal University, Anyang 455000, China

**Keywords:** colorimetric immunoassay, metal-organic framework, boronic acid, signal amplification

## Abstract

The sensitivity of immunoassays is generally limited by the low signal reporter/recognition element ratio. Nanomaterials serving as the carriers can enhance the loading number of signal reporters, thus improving the detection sensitivity. However, the general immobilization strategies, including direct physical adsorption and covalent coupling, may cause the random orientation and conformational change in proteins, partially or completely suppressing the enzymatic activity and the molecular recognition ability. In this work, we proposed a strategy to load recognition elements of antibodies and enzyme labels using boronic acid-modified metal-organic frameworks (MOFs) as the nanocarriers for signal amplification. The conjugation strategy was proposed based on the boronate ester interactions between the carbohydrate moieties in antibodies and enzymes and the boronic acid moieties on MOFs. Both enzymes and MOFs could catalyze the oxidation of 3,3′,5,5′-tetramethylbenzidine (TMB) by H_2_O_2_, therefore achieving dual signal amplification. To indicate the feasibility and sensitivity of the strategy, colorimetric immunoassays of prostate specific antigen (PSA) were performed with boronic acid-modified Cu-MOFs as peroxidase mimics to catalyze TMB oxidation and nanocarriers to load antibody and enzyme (horseradish peroxidase, HRP). According to the change in the absorbance intensity of the oxidized TMB (_ox_TMB), PSA at the concentration range of 1~250 pg/mL could be readily determined. In addition, this work presented a site-specific and oriented conjugation strategy for the modification of nanolabels with recognition elements and signal reporters, which should be valuable for the design of novel biosensors with high sensitivity and selectivity.

## 1. Introduction

Immunoassays based on the specific interaction between antigen and antibody are the gold standard in various applications, including clinical diagnosis, food quality control, and environmental monitoring [1,2]. In traditional immunoassays, the low signal reporter/antibody ratio unfavorably limits the detection sensitivity. Aiming to address this problem, nanomaterials have displayed their appropriateness and superiority in the sensitive detection of low-abundance targets by signal amplification [3]. Accordingly, the functionalized nanomaterials play versatile roles in biosensors, which can serve as the modifiers of the sensing interface to accelerate the electron transfer, nanozymes to catalyze the reactions, electrochemical or optical signal emitters, and carriers to enhance the loading number of enzymes and bioreceptors [4]. For instance, natural enzymes serving as biocatalytic labels in immunoassays have been widely integrated with nanomaterials for signal amplification, such as horseradish peroxidase (HRP), alkaline phosphatase (ALP), and glucose oxidase (GOx). Although elevating the number of enzyme labels in one immunoreaction event can promote the catalysis reaction and enhance the detection sensitivity, the immobilization of enzymes and antibodies on the solid surface may limit the enzymatic activity and the molecular recognition ability [5,6]. The general immobilization strategies, such as direct physical adsorption and covalent coupling, may cause the random orientation and conformational change of proteins, partially or completely suppressing the catalytic activity of enzymes and the binding ability of antibody and target. Therefore, site-specific and oriented conjugation strategies based on the specific recognition elements or affinity ligands are peculiarly attractive for the preparation of antibody/enzyme-modified nanolabels.

Boronic acid compounds can specifically recognize α-hydroxycarboxylate and diol-containing species by the formation of five- or six-membered cyclic boronate ester bonds. Most proteins, including antibodies and enzymes, are glycosylated with abundant carbohydrate moieties on their surface [7,8]. Thereby, a large number of boronic acid-modified nanomaterials have been utilized to specifically enrich, purify, immobilize, and label glycoproteins without affecting their biological conformation and function, such as noble metal nanoparticles, silica nanoparticles, and magnetic nanoparticles [9]. For instance, boronic acids on the surface of nanomaterials can form cyclic boronate esters with 1,2-diols on the carbohydrate moieties of an antibody to promise the correct orientation of the antibody with enhanced stability [10,11]. A variety of glycosylated enzymes, such as acetylcholinesterase, glucose oxidase, uricase, and HRP, have been immobilized on the boronic acid-conjugated biosensing interfaces, maintaining excellent biological activity for the detection of analytes or inhibitors [12,13,14,15]. Compared with other materials, metal-organic frameworks (MOFs), assembled by organic ligands and inorganic ions/clusters through the coordination interactions, possess uniform porosity and a large surface area [16,17]. The organic ligands in MOFs can provide abundant functional groups as the accessible active sites for chemical modification and immobilization of biomolecules to prevent leakage and inactivation [18,19]. Boronic acid-decorated MOFs have been successfully synthesized for the high-efficiency separation and labeling of carbohydrates [20,21,22,23]. For instance, boronic acid-functionalized, hierarchical, porous Zr-MOFs have been fabricated for the selective removal of large cis-diol-containing molecules [24]. GOx has been immobilized on the boronic acid-decorated Fe-MOFs to form integrated nanozymes for glucose detection [25].

**Scheme 1:**
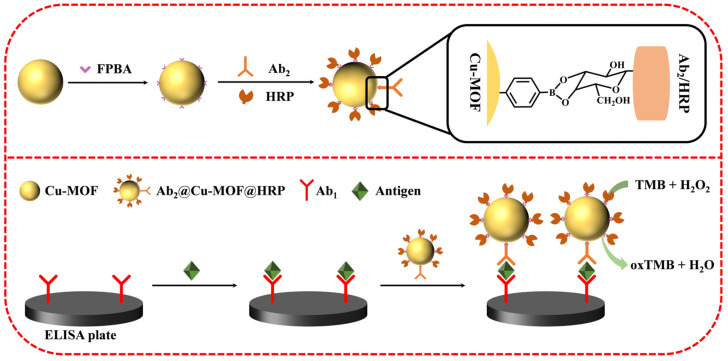
Schematic representation of sandwich-like colorimetric immunoassays with boronic acid-modified Cu-MOFs to load recognition antibody and HRP for dual signal amplification.

The diverse metal ions/clusters (e.g., Cu, Fe, Co, Ni, and Ce) with multiple valence states can serve as active sites to endow MOFs with intrinsic and tunable enzyme-like catalytic properties, such as peroxidase, oxidase, and superoxide dismutase [26,27,28]. For example, Fe and Cu-based MOFs acting as peroxidase-like nanozymes can catalyze the oxidation of chromogenic substrates (e.g., 3,3′,5,5′-tetramethylbenzidine, TMB, and o-phenylenediamine) in the presence of H_2_O_2_ for the colorimetric detection of glucose and other H_2_O_2_-generating enzyme catalysis reactions [26,27]. Ce and Co-based MOFs exhibiting oxidase-like activity can catalyze the oxidization of substrates in the absence of H_2_O_2_ [29,30]. In addition, a variety of functional materials have been integrated with MOFs to improve the catalytic performance, including metal nanoparticles, quantum dots, oxides, carbon nanomaterials, polymers, and biomolecules [31]. Among them, enzyme-linked MOF composites and MOF-based nanozymes prepared through one-pot or post-synthetic approaches have been successfully used as the integrated catalysts in various applications, such as biocatalysis, biosensing, and biomedicine. For example, Hu et al. reported a competitive electrochemical immunosensor for maduramicin detection in which an antibody and HRP were immobilized on hemin@MOFs/AuPt composites via the formation of robust Pt–N and Au–N bonds [32]. Meng et al. used HRP and antifouling material-decorated ZIF-90 as the immunoprobe to achieve the electrochemical detection of carbohydrate antigen 19-9 [33]. Zr-MOF PCN-224 has been used to simultaneously load GOx and platinum nanoparticles for the colorimetric detection of deoxynivalenol [34]. However, the immobilization of biomolecules on MOFs in these works mainly relied on the random physical or covalent interactions, which may be challenged by the complicated modification process, the low catalytic activity, and the limited molecular recognition ability in the construction of MOF-based biosensors. In this work, boronic acid-modified Cu-MOFs were employed as both the peroxidase mimics and the nanocarriers of antibody and enzyme to develop colorimetric immunoassays. The boronic acid moieties on the surface of the Cu-MOFs allowed for the well-oriented immobilization of the recognition antibody (Ab_2_) and multiple HRP molecules (Scheme 1), allowing for the molecular recognition without steric hindrance and preventing the inactivation of enzymes with improved sensitivity. More importantly, both the Cu-MOF and HRP showed high catalytic activity toward the oxidation of TMB by H_2_O_2_. The oxidized TMB (_ox_TMB) exhibited a blue color to realize the sandwich-like colorimetric immunoassays with prostate specific antigen (PSA) as a model analyte. The concentrations of PSA were sensitively determined by monitoring the changes in the absorbance and color of _ox_TMB.

## 2. Results and Discussion

### 2.1. Characterization of Cu-MOFs

**Figure 1:**
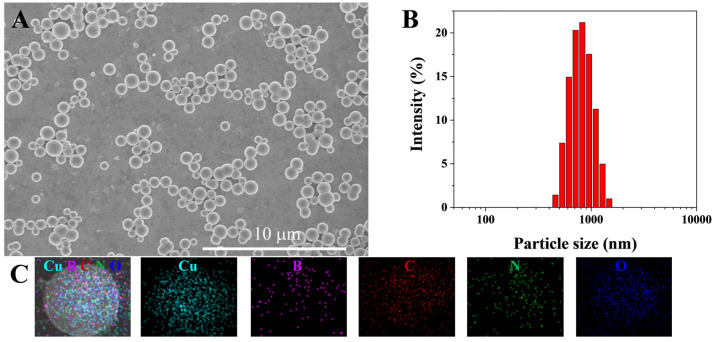
SEM image (**A**), size distribution (**B**), and EDS elemental mapping pictures (**C**) of FPBA-Cu-MOF.

**Figure 2:**
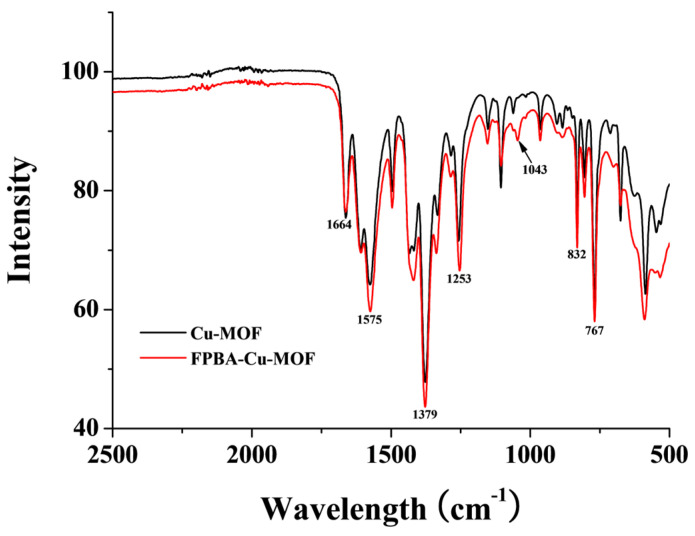
FT-IR spectra of Cu-MOF and FPBA-Cu-MOF.

Cu-MOFs with intrinsic peroxidase-like characteristics have been exploited as attractive nanozymes due to the advantages of easy synthesis, low cost, and high thermal stability and catalytic activity. The catalytic sites of Cu^2+^ ions in the frameworks can catalyze the oxidation of chromogenic substrates, thus allowing for the colorimetric detection of various analytes. In this work, Cu-MOFs were functionalized with boronic acid through the Schiff base reaction between the amino group in the ligand and the aldehyde group in FPBA [7]. The morphology of the resulting FPBA-Cu-MOF was characterized by SEM, and its average size was measured by dynamic light scattering (DLS). As depicted in Figure 1A,B, the FPBA-Cu-MOF was spherical and monodisperse, and the average size was found to be approximately 820 nm. The average size measured by DLS was larger than that observed by SEM, which is understandable since the former is derived from the hydration radius but not the true size of nanoparticles. Although no significant change in the size of the Cu-MOF was observed after the modification of FPBA, the zeta-potential changed from −19 to −28 mV. The elements of Cu, N, B, C, and O in the framework were also mapped by EDS elemental analysis (Figure 1C). To further confirm that the boronic acid moiety was modified on the MOF, Fourier-transform infrared (FT-IR) analysis was performed. As shown in Figure 2, after the modification of FPBA, an apparent new absorption peak appeared at 1043 cm^−1^ that was attributed to the absorption peak arising from BOH deformation vibration. The other absorption peaks of the boronic acid moiety were overlapped with those of the ligands, leading to an increase in the relative intensity of some peaks in the original Cu-MOF spectrum. The result is consistent with that found previously [7,20,25], suggesting that FPBA was successfully anchored on the Cu-MOF. In addition, we found that the average size of the FPBA-Cu-MOF was intensified to approximately 860 nm, and the zeta-potential of the FPBA-Cu-MOF changed into −22 mV after the modification of the antibody and HRP. This implied that the glycoproteins of the antibody and HRP could be anchored on the surface of the FPBA-Cu-MOF through the formation of boronate ester bonds.

### 2.2. Catalytic Oxidation of TMB

**Figure 3:**
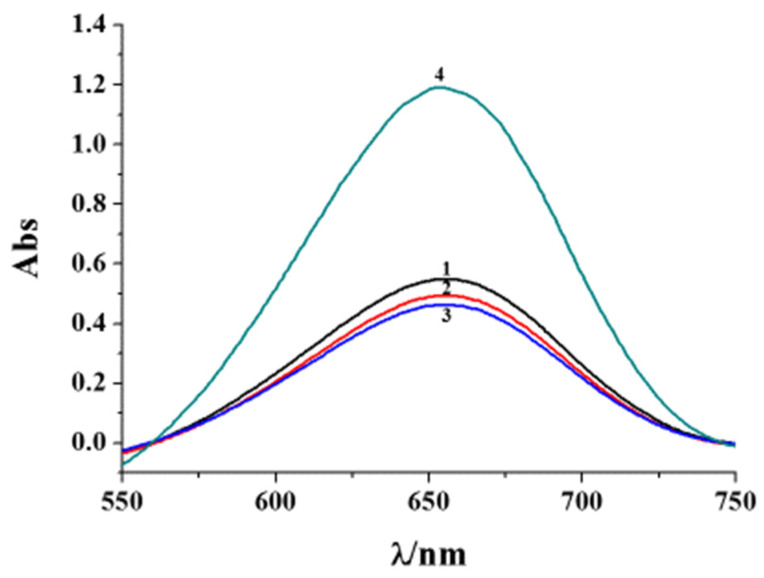
UV–vis absorption spectra of the TMB/H_2_O_2_ solution in the presence of Cu-MOF (curve 1), FPBA-Cu-MOF (curve 2), Ab_2_@Cu-MOF (curve 3), and Ab_2_@Cu-MOF@HRP (curve 4).

It has been documented that Cu-MOFs with peroxidase-like characteristics could catalyze the oxidation of TMB by H_2_O_2_ [26,27]. To investigate the effect of surface modification of diverse species on the catalytic activity of Cu-MOFs, the common substrates of TMB and H_2_O_2_ were used for the colorimetric immunoassays. After a 15 min incubation at room temperature, the UV–vis absorption spectra were collected. As shown in Figure 3, modification of FPBA on the framework did not cause a significant decrease in the absorbance intensity (c.f. curve 1 and 2), indicating that the functionalization of the Cu-MOF with boronic acid did not reduce the catalytic activity of the Cu-MOF. Note that the modification of the antibodies on the surface of the FPBA-Cu-MOF caused a negligible change in the absorbance intensity (curve 3), suggesting that the site-specific and oriented conjugation strategy based on the formation of boronate ester bonds did not limit the enzymatic activity. Interestingly, when the antibody-anchored FPBA-Cu-MOF (Ab_2_@Cu-MOF) was further modified with HRP, the absorbance intensity was remarkably intensified (curve 4), indicating that Cu-MOFs could be used as the nanocarriers of enzyme labels to enhance the catalytic performance.

### 2.3. Feasibility of Colorimetric Immunoassays

**Figure 4:**
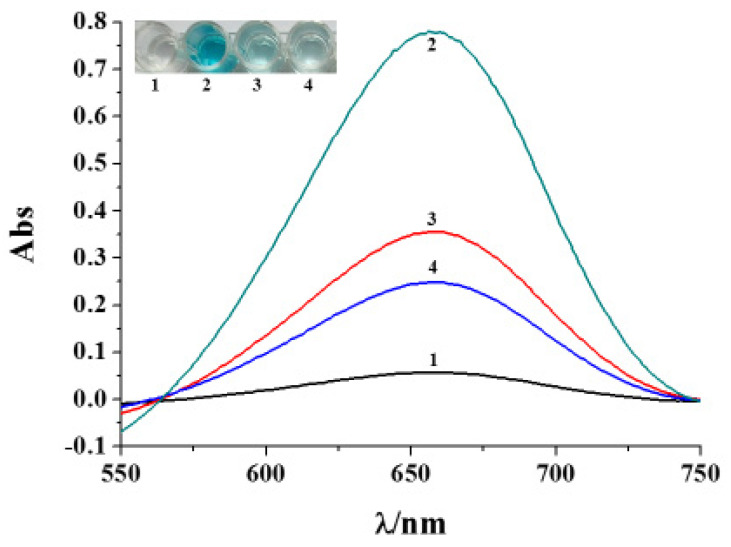
UV–vis absorption spectra and photographs of Ab_1_−coated plates after treatment by different species before the addition of TMB/H_2_O_2_: curve/plate 1, Ab_2_@Cu-MOF@HRP; curve/plate 2, PSA + Ab_2_@Cu-MOF@HRP; curve/plate 3, PSA + Ab_2_-poly-HRP; curve/plate 4, PSA + Ab_2_@Cu-MOF. The concentration of PSA was 5 ng/mL.

To investigate the feasibility of the colorimetric immunoassays, PSA, one of the recognized biomarkers for prostate diseases, was determined with the commercial ELISA plate. The capture antibody (Ab_1_) was coated on the plate surface for the capture of PSA, and the recognition antibody (Ab_2_) and HRP were modified on the FPBA-Cu-MOF as the signal label (Ab_2_@Cu-MOF@HRP). The changes in the color and absorbance intensity were monitored based on the catalytic oxidation of TMB by H_2_O_2_. As shown in Figure 4, no obvious changes in both the color and absorbance intensity were found when the Ab_1_-coated plate was incubated with Ab_2_@Cu-MOF@HRP without the capture of PSA (curve/plate 1), indicating that the signal label showed no or low non-specific adsorption on the plate. After the capture of PSA and Ab_2_@Cu-MOF@HRP, the color of the solution became blue, and the absorbance intensity was intensified (curve/plate 2). The absorbance intensity with Ab_2_@Cu-MOF@HRP as the signal label was 2.3-fold and 3.3-fold higher than that with Ab_2_-poly-HRP (curve/plate 3) and Ab_2_@Cu-MOF (curve/plate 4), respectively. This suggested that the boronic acid-modified Cu-MOF could not only serve as the peroxidase mimic to catalyze the oxidation of TMB but also act as the nanocarrier to load multiple HRP molecules for signal amplification.

### 2.4. Sensitivity

**Figure 5:**
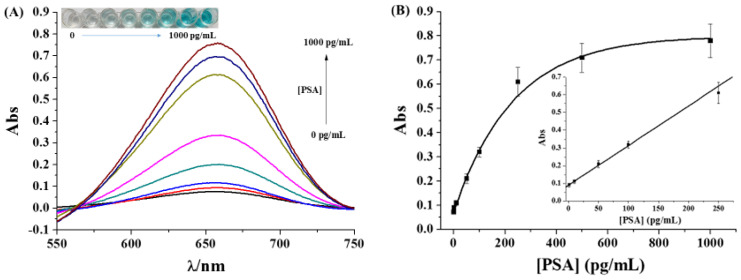
(**A**) UV–vis absorption spectra and photographs for the detection of different concentrations of PSA (0, 1, 10, 50, 100, 250, 500, and 1000 pg/mL). (**B**) Relationship between the absorbance value and PSA concentration. The inset depicts the linear fitting curve.

To study the detection performance of the method, PSA at different concentrations was analyzed. As shown in Figure 5A, with the increase in target concentration, the absorbance intensity was intensified, and the solution color became more bright blue. The absorbance value at 670 nm was linearly proportional to the target concentration in the range of 1–250 pg/mL (Figure 5B). The regression equation can be fitted as Abs = 0.089 + 0.003 [PSA] (pg/mL). The regression equation for the standard ELISA method with poly-HRP as the signal label can be fitted as Abs = 0.074 + 0.093 [PSA] (ng/mL) in the quantitative range of 0.1–2.5 ng/mL. The lowest detectable concentration of this method was at least two orders of magnitude lower than that of the ELISA method with poly-HRP as the signal label. The value for PSA detection was lower than or comparable to that with other materials as the signal labels of colorimetric immunoassays (Table 1), including enzymes or nanoenzyme-catalyzed oxidation of TMB, formation of colorful metal complexes or nanoparticles, and etching of nanomaterials. It was also comparable to that for the detection of other biomarkers, such as secreted protein acidic and rich in cysteine (SPARC) (30 fg/mL) and chloramphenicol (CAP) (0.006 μg/L) by using peptide-functionalized HRP-embedded ZIF-90 and HRP-loaded AuNP/NH_2_-MIL-101 MOFs nanocomposites as signal-amplifying tags, respectively [35,36]. The high sensitivity can be attributed to the good peroxidase-mimicking activity and the high loading capacity of the Cu-MOF. In addition, the site-specific and oriented coupling strategy for the immobilization of the antibody and enzyme through the formation of boronate ester bonds can promote the antigen–antibody recognition and maintain the catalytic activity of both the Cu-MOF and HRP.

### 2.5. Selectivity

**Figure 6:**
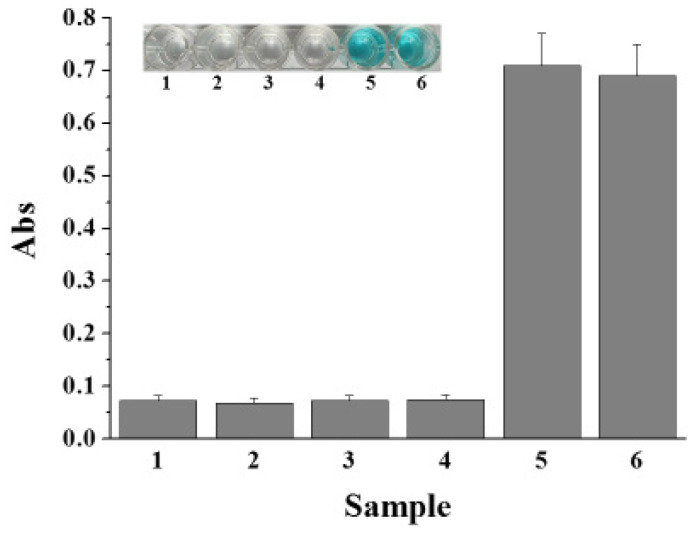
Selectivity of the immunoassay (from bar 1 to bar 6: BSA, CEA, thrombin, IgG, PSA, and the mixture of 1–5). The concentration of PSA was 0.5 ng/mL, and that of the others was 50 ng/mL.

The selectivity of the established method against the non-targets was evaluated by the cross-reactivity with other proteins at the concentration of at least 10-fold higher than that of PSA, including two common serum proteins (BSA and IgG), a cancer biomarker (CEA), and a protease (thrombin). As shown in Figure 6, the absorbance value at 670 nm was negligible, and there was no identifiable blue color for the tested non-targets of BSA, CEA, thrombin, and IgG (bars 1~4). This indicated that the immunoassay exhibited excellent selectivity due to the specific antigen–antibody interaction and the low non-specific adsorption of the signal label. Additionally, the anti-interference was challenged by determining PSA in the presence of the four non-targets. No significant differences in the solution color and absorbance intensity were observed for the target assay in the absence and presence of the non-targets (bars 5~6). The good anti-interference of the method should be attributed to the high sensitivity and selectivity of the immunoassay platform.

### 2.6. Assays of PSA in Serums

To prove the applicability of the method, three standard PSA samples were spiked in fetal bovine serum and analyzed by this method. The concentrations of PSA in the serums were determined according to the established standard curve, which are close to the added values (Table 2). The recovery rates, varying from 92.1 to 107%, are acceptable, indicating that our method possesses great potential for the detection of PSA in clinical samples. In addition, the site-specific and oriented modification of biomolecules should be valuable for the design of novel biosensors for the detection of various biomarkers in clinical applications.

## 3. Experimental

### 3.1. Regents and Instruments

4-Formylphenylboric acid (FPBA), TMB, Cu(NO_3_)_2_ hydrate, N,N-dimethylformamide (DMF), and polyvinyl pyrrolidone (PVP) were purchased from Aladdin Scientific Corp. (Shanghai, China). 2-Aminoterephthalic acid (NH_2_-BDC), bovine serum albumin (BSA), thrombin, poly-HRP, IgG, and fetal bovine serum were provided by Sigma-Aldrich (Shanghai, China). CEA and PSA kits were ordered from Linc-Bio Science Co. Ltd. (Shanghai, China). HRP was obtained from Sangon Biotech. Co., Ltd. (Shanghai, China). Other chemical reagents were of analytical grade and used without additional purification.

The UV–vis spectra were collected on a Cary 60 spectrophotometer using a quartz spectrophotometer cell. The morphologies of MOFs were characterized by a SU8010 scanning electron microscopy (SEM, Hitachi, Japan). The average size and Zeta potentials of MOFs in phosphate buffer (10 mM, pH 7.4) were recorded on a Malvern Zetasizer Nano ZS model ZEN 3600 (Worcestershire, UK) at 25 °C with 12 zeta runs. The ultrapure water used in the experiments was prepared by a Milli-Q purification system. FT-IR spectra were collected on a Nicolet iS10 spectrophotometer (Thermo Scientific, Inc., Waltham, MA, USA).

### 3.2. Preparation of Boronic Acid-Modified Cu-MOFs

The diverse redox metal ions/clusters with multiple valence states can serve as the active sites to endow MOFs with intrinsic and tunable enzyme-like catalytic properties. Typically, Fe and Cu-based MOFs have been used as peroxidase-like nanozymes to catalyze the oxidation of the chromogenic substrate TMB in the presence of H_2_O_2_ for the colorimetric assays. In this work, Cu-MOFs were used to catalyze the oxidation of TMB and serve as the nanocarriers to load the antibody and HRP. The method for the preparation of Cu-MOFs followed that in the previous report [7]. Briefly, 0.2 g of PVP was dissolved in a mixture of 4 mL DMF and 4 mL ethanol (solution A). Both 24.2 mg of Cu(NO_3_)_2_ hydrate and 5.4 mg of NH_2_-BDC were dissolved in 4 mL DMF (solution B). The A and B solutions were then mixed under ultrasonication for 30 min. After that, the mixed solution was heated in a Teflon-lined autoclave at 100 °C for 8 h. After centrifugation at 5000 rpm for 10 min, the precipitates were collected, washed with DMF and ethanol several times, and then dried in a vacuum oven at 60 °C overnight.

Boronic acid can specifically react with diol-containing species by the formation of boronate ester bonds. Natural antibodies and some enzymes are glycosylated with abundant carbohydrate moieties on their surface, which allows for the enrichment, purification, immobilization, and labeling of antibodies and enzymes with boronic acid-modified substrates or materials. In this work, FPBA molecules were conjugated onto the prepared Cu-MOF with the previously reported procedure [7]. In brief, 6 mg of Cu-MOF powder was dissolved in 6 mL ethanol under ultrasonication for 10 min. Then, 6 mg of FPBA was added to the solution. The mixture was refluxed at 80 °C for 12 h under continuous stirring. The resulting FPBA-Cu-MOF precipitates were collected by centrifugation at 5000 rpm for 10 min, washed with ethanol three times, and then dried at 60 °C overnight under vacuum.

### 3.3. Functionalization of FPBA-Cu-MOF with Antibody and HRP

PSA antibody (Ab_2_) and HRP were anchored on the surface of the FPBA-Cu-MOF through the formation of boronate ester bonds. In brief, 0.2 mg of FPBA-Cu-MOF solid was dispersed in 1 mL phosphate buffer (10 mM, pH 7.4). After sonication for 5 min, 10 μL of Ab_2_ (1 μg/mL) solution was added to the FPBA-Cu-MOF suspension. After gentle shaking for 10 min, 10 μL of HRP (0.1 mg/mL) solution was added to the above suspension. After washing three times by centrifugation with the phosphate buffer to remove the free Ab_2_ and HRP, the resulting Ab_2_@Cu-MOF@HRP was dispersed in 10 mL phosphate buffer for use.

### 3.4. Colorimetric Immunoassays

The colorimetric immunoassays were conducted by using a PSA antibody (Ab_1_)-coated ELISA plate. In brief, 100 μL of PSA sample or the tested non-target protein was spiked in the ELISA plate to incubate for 1 h at room temperature. After washing the plate with 200 μL buffer three times, 100 μL of the prepared Ab_2_@Cu-MOF@HRP suspension was added to the plate. After incubation for 1 h again, the plate was rinsed with 200 μL of buffer. Then, 100 μL of reaction buffer containing 0.5 mM TMB and 50 mM H_2_O_2_ was successively added to each plate. After incubation for 15 min, the UV absorbance spectra were recorded on a Cary 60 UV–vis spectrophotometer, and meanwhile, the photographic images were collected. To demonstrate the practicality of the proposed method, three PSA standard samples were spiked in 10% fetal bovine serum and then analyzed by the same aforementioned procedure. The concentrations of PSA were determined according to the established calibration method.

## 4. Conclusions

In summary, a site-specific and oriented conjugation strategy was reported for the preparation of antibody/enzyme-modified Cu-MOF nanolabels. In contrast to other immobilization strategies, such as direct physical adsorption and covalent coupling, the proposed modification method is simple and does not require complex cross-linking procedures. More importantly, the site-specific and oriented immobilization of antibody and enzyme through the carbohydrate moieties did not suppress the catalytic activity of the enzymes and the binding ability of the antigen–antibody. Benefiting from the controllable conjugation and the high catalytic efficiency of both the Cu-MOF as well as HRP, the dual signal amplification colorimetric immunoassays exhibited high sensitivity and selectivity. The lowest detectable concentration of 1 pg/mL is comparable to or even lower than that determined by other immunoassays. This work should be valuable for the site-specific and oriented modification of biomarkers and the development of novel biosensors for the detection of various targets.

## Figures and Tables

**Table 1:** Analytical performances of different strategies for colorimetric immunoassays of PSA.

Chromogenic Reaction	Signal Label	Linear Range (pg/mL)	LOD (pg/mL)	Ref.
TMB oxidation	Au NPs	2.5 × 10^2^–2.5 × 10^6^	230	[37]
TMB oxidation	a-RuTe_2_	50–5 × 10^3^	32.6	[38]
TMB oxidation	AuNPs-catalase	50–2 × 10^4^	30	[39]
TMB oxidation	AuNC/GNRs	10–2 × 10^3^	10	[40]
TMB oxidation	HRP@PCN-333	15–1.65 × 10^2^	6	[41]
TMB oxidation	Au@PtNHs	5–5 × 10^2^	2.9	[42]
TMB oxidation	Fe_SA_−Pt_C_	8–1 × 10^3^	1.8	[43]
TMB oxidation	PdSn nanozymes	2–2 × 10^3^	1.696	[44]
TMB oxidation	GOx@FeCPs	10–2 × 10^3^	1.03	[45]
TMB oxidation	Cu-P NPs	1–10^3^	1	[46]
TMB oxidation	PDA-Fe^3+^ NPs	0.5–2 × 10^4^	0.87	[47]
TMB oxidation	HIF-Pt-CNCs	20–2 × 10^3^	0.8	[48]
TMB oxidation	MY/ZIF-8	1–1 × 10^3^	0.67	[49]
TMB oxidation	IL-Cu@Cu_2_O	5–1 × 10^3^	0.63	[50]
TMB oxidation	AgNPs	2 − 64	0.165	[51]
Cu(I)-BCA complex	ALP	5×10^2^ − 2.5 × 10^4^	380	[52]
Iron(III) complex	AuNP-GOx	1–3 × 10^4^	0.5	[53]
Iodine–starch complex	GOx	1–10^6^	0.46	[54]
Formation of Si NPs	ALP	20 − 2.8 × 10^4^	9.6	[55]
Growth of Au on PDA	ALP	50–1 × 10^5^	6.71	[56]
AuNPs growth	ALP	1–2 × 10^2^	3 × 10^−2^	[57]
AuNPs growth	MB-GOx	1 × 10^−2^–1 × 10^2^	3.1 × 10^−3^	[58]
AuNPs aggregation	GOx	0–10^4^	4 × 10^3^	[59]
Etching of AuNRs	HRP	Not reported	75	[60]
Etching of AgNPRs	GOx	0.01–1 × 10^2^	4.1 × 10^−3^	[61]
pH indicator	TP-MSN	0.5–8 × 10^3^	0.36	[62]
pH indicator	GSH-AuNPs	1 × 10^2^–10^4^	1 × 10^2^	[63]
4-NP reduction	Au/Bi_2_Se_3_ NSs	Not reported	72	[64]
Fe(III)-ferrozine/TCEP	MSN/PQQ	5–5 × 10^3^	1	[65]
TMB oxidation	Poly-HRP	1 × 10^2^–2.5 × 10^3^	1 × 10^2^	This work
TMB oxidation	MOF-HRP	1–2.5 × 10^2^	1	This work

Abbreviation: AuNPs, gold nanoparticles; IL-Cu@Cu_2_O, ionic liquid functionalized Cu@Cu_2_O aerogels; PL-CsPbBr_3_, phospholipid-coated CsPbBr_3_; HRP, horseradish peroxidase; Fe_SA_−Pt_C_, Pt clusters on the Fe single-atom nanozymes; a-RuTe_2_, amorphous RuTe_2_ nanorods; PDA, polydopamine nanoparticles; GOx@FeCPs, Fe(III)-based coordination polymers to simultaneously integrate glucose oxidase; MY/ZIF-8, methyl yellow-loaded ZIF-8 metal-organic frameworks; AgNPs, silver nanoparticles; HIF-Pt-CNCs, high-index {*hk*0} faceted platinum concave nanocubes; Au@PtNHs, (gold core)@(platinum shell) nanohybrids; GNRs, gold nanorod composites; Cu-P NPs, biotinylated peptide-Cu^2+^ nanoparticles (Cu-P NPs); GOx, glucose oxidase; ALP, alkaline phosphatase; BCA, bicinchoninic acid; AgNPRs, triangular silver nanoprism; TP, thymolphthalein; MSN, mesoporous silica nanoparticle; GSH, glutathione; NSs, nanosheets; 4-NP, 4-nitrophenol; TCEP, tris(2-carboxyethyl)phosphine; PQQ, pyrroloquinoline quinone.

**Table 2:** Results for the assays of PSA spiked in serums.

Added (pg/mL)	Found by This Method (pg/mL)	**Recovery Rate (%)**
10	10.7	107
50	49.2	98.4
100	92.1	92.1

## Data Availability

Data are contained within the article.
